# Stepping into the real world: a mixed-methods evaluation of the implementation of electronic patient reported outcomes in routine lung cancer care

**DOI:** 10.1186/s41687-022-00475-6

**Published:** 2022-06-20

**Authors:** Afaf Girgis, Adeola Bamgboje-Ayodele, Orlando Rincones, Shalini K. Vinod, Sandra Avery, Joseph Descallar, Allan ‘Ben’ Smith, Belinda Arnold, Anthony Arnold, Victoria Bray, Ivana Durcinoska, Nicole M. Rankin, the PROMPT-Care Implementation Authorship Group, Chee Fon Chang, Bianka Eifler, Sarah Elliott, Christine Hardy, Beth Ivimey, William Jansens, Nasreen Kaadan, Eng-Siew Koh, Nic Livio, Susan Lozenkovski, Gemma McErlean, Elias Nasser, Nicola Ryan, Therese Smeal, Tien Thomas, Thomas Tran, Jennifer Wiltshire, Geoff P. Delaney

**Affiliations:** 1 South West Sydney Clinical Campuses, UNSW Medicine and Health, University of New South Wales, Sydney, NSW 2052 Australia; 2 Ingham Institute for Applied Medical Research, Liverpool, NSW 1871 Australia; 3 School of Medical Sciences, Biomedical Informatics and Digital Health, Faculty of Medicine and Health, The University of Sydney, Sydney, NSW 2006 Australia; 4 Liverpool Cancer Therapy Centre, Liverpool Hospital, South Western Sydney Local Health District, Liverpool, NSW Australia; 5 Macarthur Cancer Therapy Centre, South Western Sydney Local Health District, Campbelltown, NSW 2560 Australia; 6 Bankstown Cancer Therapy Centre, South Western Sydney Local Health District, Bankstown, NSW 2200 Australia; 7 Wollongong Hospital, Illawarra Shoalhaven Local Health District, Wollongong, NSW Australia; 8 Sydney School of Public Health, The University of Sydney, Sydney, NSW 2006 Australia

## Abstract

**Background:**

To realize the broader benefits of electronic patient-reported outcome measures (ePROMs) in routine care, we used the RE-AIM (Reach, Effectiveness, Adoption, Implementation, and Maintenance) framework to inform the translation of a clinically effective ePROM system (hereafter referred to as the PRM system) into practice. The study aimed to evaluate the processes and success of implementing the PRM system in the routine care of patients diagnosed with lung cancer.

**Method:**

A controlled before-and-after mixed-methods study was undertaken. Data sources included a self-report questionnaire and interviews with healthcare providers, electronic health record data for PRMs patients and historical controls, and field notes. Descriptive statistics, logistic regression modelling, negative binomial models, generalized estimating equations and repeated measures ANOVA were used to analyze quantitative data. Qualitative data was thematically analyzed.

**Results:**

A total of 48/79 eligible people diagnosed with lung cancer completed 90 assessments during the 5-month implementation period (RE-AIM *reach*). Every assessment breached the pre-defined threshold and care coordinators reviewed and actioned 95.6% of breaches, resulting in 146 referrals to allied health services, most frequently for social work (25.3%), dietetics (18.5%), physiotherapy (18.5%) and occupational therapy (17.1%). PRMs patients had significantly fewer visits to the cancer assessment unit for problematic symptoms (M = 0.23 vs. M = 0.43; *p* = 0.035), and were significantly more likely to be offered referrals (71% vs. 29%, *p* < 0.0001) than historical controls (RE-AIM *effect*). The levels of ‘organizational readiness for implementing change’ (ORIC) did not show much differences between baseline and follow-up, though this was already high at baseline; but significantly more staff reported improved confidence when asking patients to complete assessments (64.7% at baseline vs. 88.2% at follow-up, *p* = 0.0046), and when describing the assessment tool to patients (64.7% at baseline vs. 76.47% at follow-up, *p* = 0.0018) (RE-AIM *adoption*). A total of 78 staff received PRM system training, and 95.6% of the PRM system alerts were actioned (RE-AIM *implementation*); and all lung cancer care coordinators were engaged with the PRM system beyond the end of the study period (RE-AIM *maintenance*).

**Conclusion:**

This study demonstrates the potential of the PRM system in enhancing the routine care of lung cancer patients, through leveraging the capabilities of automated web-based care options.

**Plain English summary:**

Research has shown the clear benefits of using electronically collected patient-reported outcome measures (ePROMs) for cancer patients and health services. However, we need to better understand how to implement ePROMs as part of routine care. This study evaluated the processes and outcomes of implementing an ePROMs system in the routine care of patients diagnosed with lung cancer. Key findings included: (a) a majority of eligible patients completed the scheduled assessments; (b) patient concerns were identified in every assessment, and care coordinators reviewed and actioned almost all of these, including making significantly more referrals to allied health services; (c) patients completing assessments regularly were less likely to present to the cancer assessment unit with problematic symptoms, suggesting that ePROMs identified patient concerns early and this led to a timely response to concerns; (d) staff training and engagement was high, and staff reporting increased confidence when asking patients to complete assessments and when describing the assessment tool to patients at the end of the implementation period. This study shows that implementing ePROMs in routine care is feasible and can lead to improvements in patient care.

**Supplementary Information:**

The online version contains supplementary material available at 10.1186/s41687-022-00475-6.

## Introduction

Lung cancer is the leading cause of cancer death globally [1]. Despite improved survival and prognosis, the physical and psychosocial issues which appear early in the cancer journey often persist for patients who move into long-term survivorship, and often precipitate unnecessary hospitalization and significant individual and health system burden [2, 3].

The anticipated increased demand for cancer services, plus the COVID-19 pandemic, highlight the need for flexible patient care. Telehealth, web-based care and long-term follow-up are potentially viable alternative/complementary models of care for this growing demand [4–6]. Remote web-based patient-reported outcome measures (ePROMs) facilitate patients reporting issues of concern to their care team, thereby may prompt timely care according to level of need [7, 8].

Health care services are increasingly incorporating ePROMs to inform person-centred care and evaluate services [9]. In the research context, well-integrated ePROM systems are demonstrated to be acceptable and feasible to implement [10] with improved patient and health system outcomes, including patient-provider communication, patient satisfaction [11], health-related quality of life [12, 13], compliance with chemotherapy [13]; earlier detection of relapse in lung cancer patients [14]; reduced emergency department (ED) presentations [15–17]; and improved cancer survival [16, 18].

In 2013, our team developed an ePROM system, PROMPT-Care (Patient Reported Outcome Measures for Personalized Treatment and Care) and demonstrated its acceptability, feasibility [10], and impact on ED presentations [17]. This evidence provided the impetus for its implementation in routine care. However, despite good evidence of ePROMs’ effectiveness in improving patient and health service outcomes and some guidelines available [19–21], implementation in routine clinical care remains challenging; and internationally, there are few examples to guide large-scale ePROM implementation in oncology, with Cancer Care Ontario being a noteworthy exception [22].

Implementation science can inform the translation of clinically effective interventions, such as ePROMs, into practice [23]. The identified potential barriers and facilitators to implementing ePROMs in health services [9, 23–25] suggest a strong need for organizational preparedness through stakeholder engagement and organizational resourcing. Recommended strategies include communicating the robustness and value of collecting PROMs, integrating PROMs into the electronic medical record (eMR), training clinicians in using them, integrating ePROM collection into clinical workflows, and technical support and feedback mechanisms in clinics [9, 24, 25].

This study aimed to evaluate the processes and success of implementing our ePROM system in the routine care of patients diagnosed with lung cancer.

## Methods

Ethics and governance approval was received from the South Western Sydney Local Health District (SWSLHD) ethics committee, Ref. 2020/ETH01052. Standards for reporting implementation studies of complex interventions (StaRI) guidelines [26] were followed.

### Design

A controlled before-and-after mixed-methods study was undertaken to evaluate the implementation of the new model of care.

### Implementation context and setting

The implementation context for ePROMs was influenced by the socio-economic environment of participating hospitals, which is known as a socially, economically, culturally, and linguistically diverse population, with a lot of its population born overseas, and almost half of its population speaking a language other than English at home (as previously described [27]). This study was conducted in (SWSLHD), a metropolitan area in Sydney, Australia. SWSLHD comprises six hospitals, serving more than 966,000 people (12% of residents in New South Wales (NSW), Australia’s most populous state). The area contributes 10% of total new cancer cases in NSW [28]. Participating hospitals had care coordinator resources, access to specialist lung cancer clinicians and treatments, and previous PROMPT-Care system experience.

### The ePROM (PRM) system

The PROMPT-Care Version 2 ePROM system, previously described [29], is fully integrated into the patients’ eMR and supports patient management through (a) monthly physical and psychosocial wellbeing ePROM using the Distress Thermometer (DT) and associated checklist [30], and the Edmonton Symptom Assessment Scale (ESAS) [31], (b) automated email clinical alerts notifying the care team of unresolved clinical issues, and (c) tailored patient self-management resources to help them meet their identified needs and concerns. The Cancer Institute NSW (the Government-funded state-wide cancer control agency) adapted Version 2, including automating much of its functionality. This adapted Version 3, the system implemented in routine care in SWSLHD, is hereafter referred to as the PRMs system. The key changes on the latest version of the PRMs system were: (a) the system being hosted and integrated by the state-wide government-funded cancer authority (the CINSW) and not the individual local health districts, (b) deleting the 9-item Supportive Care Needs Survey-Screening Tool 9, (c) patients being able to elect receipt of surveys via email or text message (SMS), (d) incorporation of greater privacy measures (e.g. a 2-step device authentication, including acceptance to receive or decline surveys).

### Implementation framework

We adopted the RE-AIM framework to guide implementation. Its five domains are: (a) *reach* of the target population; (b) *effect* on key outcomes; (c) *adoption* by people responsible for its delivery; (d) success of its *implementation*; and (e) potential for it to be *maintained* [32].

### Participants

#### Health care professionals (HCPs)

Staff at participating hospitals involved in lung cancer care delivery, including but not limited to administrative, specialist, nursing and allied health staff, had potential involvement in implementation.

#### Patients

All eligible patients were introduced to PRMs screening as part of their routine care, if they: (a) had a confirmed diagnosis of lung cancer, (b) were receiving care in a participating hospital, (c) were able to complete (themselves or with assistance) the PRMs assessment in English, and (d) were not currently participating in a clinical trial.

Only de-identified patient data was required for evaluation purposes. Data were extracted from the eMR, providing general data on ED presentations, and the oncology information system (OIS), providing data related to diagnosis, cancer assessment unit (CAU), and medical and radiation oncology. The CAU is where unwell cancer patients can present for assessment and management by an experienced nurse and oncology trainees to prevent ED presentation and hospital admissions.

### Procedure

During their first post-diagnosis clinic appointment, patients were informed that they would complete PRMs assessments regularly as part of their routine care. In clinic, care coordinators provided patients with an iPad to complete their first assessment, with subsequent assessments completed either in-clinic or remotely from home at a frequency determined by each participating hospital. Care coordinators reviewed patients’ PRMs results generated in the eMR and responded to automated email clinical alerts identifying patients with high levels of psychosocial distress or physical symptoms, offering interventions or referrals as necessary.

### Implementation plan

The implementation plan is described elsewhere [33]. A multidisciplinary implementation advisory group was formed, with representation from participating hospitals, to identify local champions to adapt and incorporate the PRMs model of care into the local contexts. Stakeholders decided on patient eligibility (lung cancer selected for initial roll-out), onboarding PRMs processes, and follow-up and referral pathways at planning meetings. Processes had to align with existing workflows, use existing staff resources, minimize staff and patient burden, and maximize onboarding. Implementation planning occurred from October 2019 to November 2020, GO-Live for the enhanced PRMs system was achieved on 16th November 2020, and evaluation occurred five months later. The period of implementation spanned the COVID-19 outbreak, and included multiple periods of local community lockdowns. This had significant effects on the models of care of the entire cancer service and the PRMs implementation programme, including using much more teleconferencing and much less face to face discussion. Some cancer care coordinators preferred conducting the initial onboarding of patients via phone consultations beyond the COVID-19 outbreak. The implementation group met regularly to problem shoot during this period.

### Historical controls

The historical control group included new lung cancer patients seen at participating centres between mid-November 2018 and mid-April 2019, coinciding with the 5-month implementation evaluation period, in the year immediately preceding the COVID-19 pandemic. For comparability between the PRMs and control cohorts, clinicians screened control patients to ensure eligibility matched the PRMs patients.

### Data collection sources

#### Clinic audit logs

We conducted a pre-implementation clinic audit to determine the proportion of patients with access to out-of-clinic internet; preferring to complete PRMs assessments on paper versus electronically; capable of completing assessments in English; and anticipating requiring assistance to complete assessments. This audit included patients with all cancer types and from hospitals in SWSLHD and elsewhere, as reported previously [27]. This paper only reports on the results of the lung cancer sub-group of the published audit.

#### HCP Survey

Invitations to complete a baseline online survey were emailed to HCPs who participated in any workshops, meetings, orientation and training regarding PRMs system implementation between June and November 2020. Five months after the GO-Live date, a subsequent invitation to complete an online follow-up survey was emailed to those who completed the baseline survey. Both the baseline and follow-up surveys included the Organizational Readiness for Implementing Change (ORIC) scale and HCP survey (see below). The follow-up survey included additional questions regarding HCPs’ engagement with the PRMs reports.

The ORIC survey [34] comprises 12-items and two subscales—change commitment (a shared resolve to implement a change within an organization), and change efficacy (collective capability to implement a change). Higher scores (range 12–60) indicate greater organizational readiness for change. The scale has strong psychometric properties and has been validated in real-world hospital settings [35].

The HCP survey was adapted from a previously published measure [36], examining staff and environmental factors related to successful implementation of interventions in hospital settings [37]. The 27-item questionnaire (Additional file 1) assessed barriers (8-items), knowledge and attitudes (4-items), HCP confidence and role (6-items); and demographic and workplace characteristics (9-items); and included a free-text question on barriers.

#### Staff interviews

At study completion, 11 HCPs and OIS staff were purposefully selected to provide feedback on the PRMs system in semi-structured virtual interviews. Staff were selected to represent the continuum of activities across the PRMs system, including system infrastructure and IT support (n = 2), onboarding patients, reviewing PRMs results and coordinating patient care (n = 3 care coordinators), oncology care (n = 2 medical, and n = 3 radiation oncologists), and response to allied health referrals (n = 1 social worker). Depending on their specialty, participants were asked about aspects of implementation including what worked well and did not, strategies for improving clinician engagement, and recommendations for system enhancements before wider-scale implementation with other tumour groups.

#### HCP training log

A log was maintained of the number of HCPs participating in training, including completing orientation and receiving the implementation resources/toolkit.

#### Patient health records

OIS staff extracted patient demographic, clinical, and health services data; and eMR staff extracted data on the use of the CAU and ED presentations.

#### Field notes

Stakeholder meetings and HCP workshops were audio-recorded and monitored via field notes, as detailed elsewhere [33].

### Outcomes

Table 1 details how specific components of the PRMs system implementation were evaluated based on the RE-AIM domains. To evaluate the implementation process: (1) baseline data were extracted for pre-GO-Live and follow-up data five months later (*Reach, Adoption, Maintenance*); (2) data were collected throughout implementation (*Reach, Adoption, Implementation, Maintenance*); and (3) historical control data were compared with implementation data throughout implementation (*Effect*).

**Table 1:** Components of RE-AIM evaluation framework

Constructs and definitions applied for this study	Questions addressed	Data sources used (# aligns with the # for each question addressed)	Timepoint
*Reach*	1. What is the number and proportion of patients in clinic	1. Clinical audit log completed over a 3-month period	Baseline (pre-implementation)
The number, proportion and representativeness of people who attend lung cancer clinic	(a) with internet/mobile phone access at home		
	(b) capable of completing assessment in English vs other native language		
	(c) who require assistance to complete assessments		
	(d) who prefer to complete PROs on paper vs electronic device?		
	2. What is the number and proportion of patients completing assessment screening including the number and proportion of those systemically missed and why?	2. Oncology Information System (OIS)—MOSAIQ records	At first assessment timepoint in clinic
*Effectiveness*	Compared to a historical control cohort:	1. Electronic medical records (eMR)	Historical control and throughout implementation period
The effectiveness of ePROs in improving lung cancer care	1. What is the impact of ePROs on the number of admissions to hospital due to ED presentations?		
	2. What is the impact of ePROs on the use of the cancer assessment unit?		
	3. What is the impact of ePROs on care coordinator referral rates?	2 and 3. OIS (MOSAIQ) records	
*Adoption*	1. What is the extent to which the new model of care and implementation strategies are considered feasible and appropriate among HCPs, given the different needs and resources of each hospital?	1 and 2. Organizational readiness for implementing change (ORIC) survey	Baseline and at 5-months
The development of organizational support to deliver ePROs			
	2. What is the number, proportion and representation of HCP (change agents) implementing ePROs?	1 and 2. ePROM HCP Survey	Baseline and at 5-months
	3. What is the number and proportion of all cancer services staff completing ePROM orientation?	1. Field notes and recordings of HCP feedback during MDT meetings and workshops	Throughout implementation period
		2 and 3. Orientation attendance logs	
*Implementation*	1. What is the number and proportion of patients screened at each hospital?	1 and 2. OIS (MOSAIQ) records	Throughout implementation period
The extent of ePROM implementation at each hospital. i.e. the degree to which each hospital receives the planned implementation strategies, and success of implementation processes			
	2. What is the number and proportion of clinical alerts actioned or followed up by HCP?		
	3. What is number of HCPs receiving orientation and implementation resources?	3. Orientation attendance logs	
		3. PROMs website views	
*Maintenance*	1. What is the number and proportion of staff who maintain ePROM engagement over a 5-month implementation?	1. HCP survey (baseline and follow-up)	Baseline and follow-up
Incorporating ePROs in routine care to ensure sustainability over time			
	2. What is staff feedback on ePROs implementation in routine clinical practice?	1. OIS (MOSAIQ) records	Throughout implementation period
		2. Interviews with HCP and field notes	Post-implementation

### Analysis

Quantitative data collected via audits, logs and surveys were analyzed using descriptive statistics including means (and Standard Deviations (SD)), frequencies, and proportions. Multivariable logistic regression modelling was used to analyze the number of above-threshold breaches (< 13 vs. ≥ 13; 13 being the average number of items breached across all assessments), ED presentations (≥ 1 visit vs. no visits), CAU visits (≥ 1 visit vs. no visits), and referral acceptance (yes/no). Negative binomial models were used to analyze the numbers of ED presentations, hours hospitalized due to ED presentations, CAU presentations, and referred patients. ED presentations were also compared against the group of patients who declined PRM assessments. Generalized estimating equations (GEE) were used to analyze the likelihood of referral acceptance for those breaching a high number of items. Repeated measures ANOVA was used to analyze ORIC scores over time. Statistical analysis was performed in SAS Enterprise Guide version 8.2.

Qualitative data from open-ended survey responses, field notes and interviews were thematically analyzed [38]. Staff interviews were audio-recorded, transcribed verbatim, and analyzed, with two researchers (AG, OR) independently reading the transcripts and generating initial codes. Identified codes were collated into emerging themes and refined, resolving discrepancies through discussion and consensus. This occurred throughout implementation and post-implementation.

## Results

### Reach

The number, proportion and representativeness of patients who attended lung cancer clinics was assessed [27]. Of the 64 audited patients, 54.7% (35/64) reported having internet/mobile phone access; 34.4% (22/64) had email access; and 68.7% (44/64) felt capable of completing assessments in English, with the top three other languages nominated being Vietnamese (27.3%, 6/22), Serbian (13.6%, 3/22) and Arabic (9.1%, 2/22). Almost half (45.3%; 29/64) of the audited patients reported requiring assistance to complete assessments; and 57.8% (37/64) expressed a preference for paper assessments, 28.1% (18/64) using a device and 6.2% (4/64) either.

**Fig. 1:**
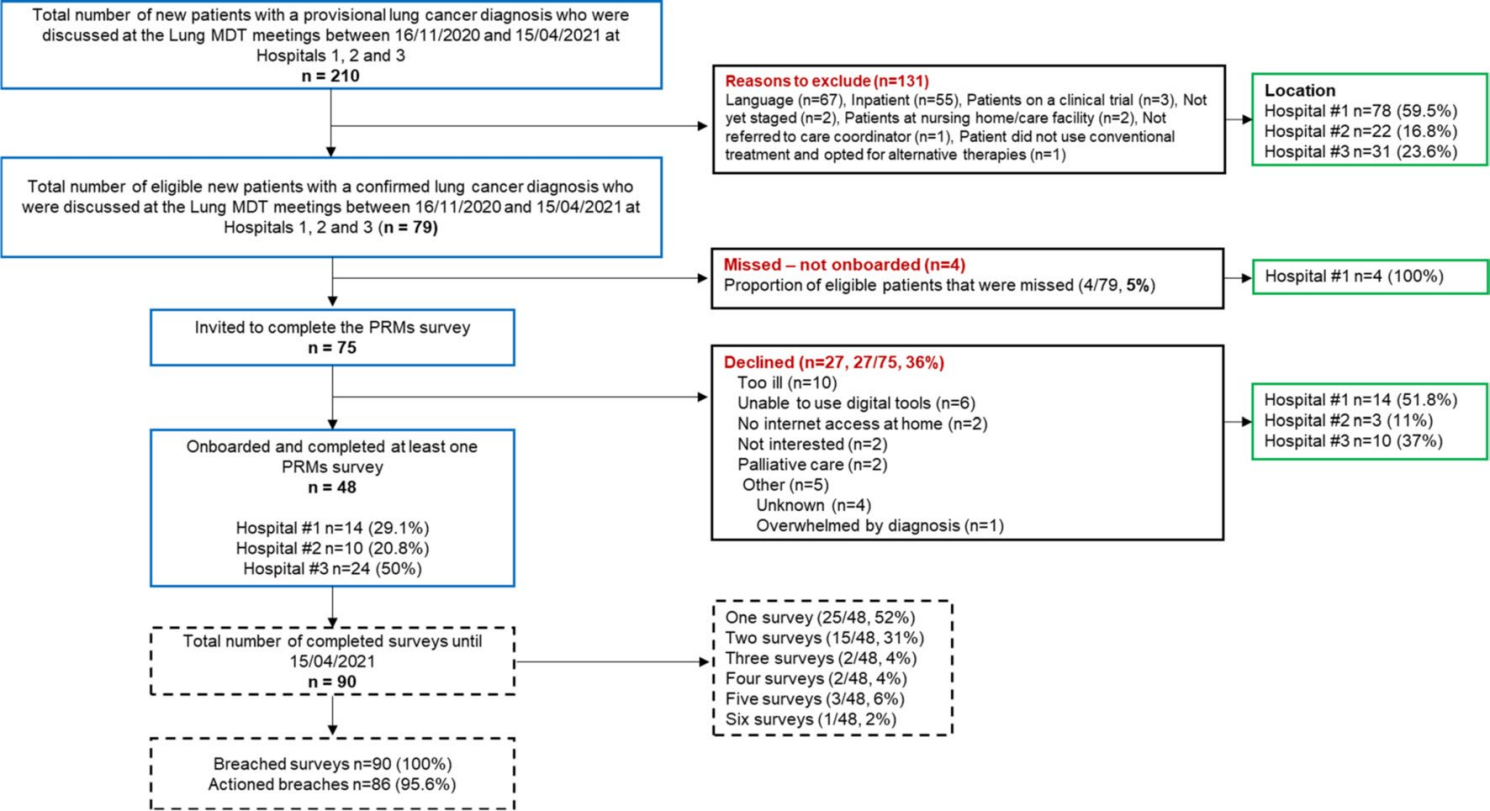
Study diagram

As shown in the CONSORT diagram (Fig. 1), of the 79 eligible new lung cancer patients, 95% (n = 75) were screened during the 5-month evaluation period, and 61% (n = 48) of the target population was onboarded, i.e. *reached* by implementation efforts. Out of 75 screened patients, 27 (36%) declined (see Fig. 1 for reasons). Reasons why some patients (5%, n = 4) were systematically missed at Hospital #1 are unknown. Characteristics of onboarded patients are shown in Table 2 (n = 48).

**Table 2:** Characteristics of onboarded patients

Characteristics	Values
Age (years), mean (SD) [Range]	69 (9) [42–85]
Sex	n (%)
Male	29 (60.4)
Female	19 (39.6)
Stage of disease	
I	4 (8.3)
II	5 (10.4)
III	11 (22.9)
IV	24 (50)
Unknown	4 (8.3)
Treatment received	
Chemotherapy*	37 (77)
Radiotherapy*	35 (72.9)
Surgery	7 (14.6)
Immunotherapy*	19 (39.6)

Note 1: patients received multiple treatments

*Started treatments; most were completed after the trial period end date 15/04/2021

Although participants were expected to complete an assessment on a monthly basis, the patient-centred approach of the cancer service meant that some patients were scheduled to complete assessments more or less frequently, depending on the stage of their cancer journey, treatment plan and personal preferences (e.g. some participants preferred to complete assessments less frequently). For these reasons, approximately half of the participants (54.1%, n = 26) completed only one assessment during the evaluation period. Of n = 12 who were due for a second assessment, only n = 2 declined to complete it (4% of the whole sample), with other reasons for non-completion including patient deceased (n = 4, 8%) or technical reasons for assessment not being sent (n = 7, 14%).

### Effect

#### Assessment breaches

The 48 PRMs patients all completed at least one assessment, with a total of 90 assessments completed overall. Every assessment resulted in at least one breach (100% breach rate), with 13.4 out of 49 items (27%) breached on average. The three most breached items were worry (65.6%), tiredness (64.4%) and fatigue (61.1%). Multivariable logistic regression analysis revealed that patients breaching ≥ 13 items were younger (*p* = 0.002) and more likely to be female (*p* = 0.004) than patients breaching < 13 items. Stage of disease did not affect the number of items breached.

There were no significant differences in gender, age or stage of disease between the PRMs group (n = 48), patients who declined onboarding (n = 27) and historical controls (n = 63).

#### ED presentations

No between-group differences were detected in the number of patients with ≥ 1 ED presentation (multi-variable logistic regression; PRMs 58.3%, 28/48; Controls 60.3%, 38/63; Declined 63%, 17/27); in the number of hours hospitalised due to ED presentations, controlling for age, gender and stage (multivariable negative binomial regression; PRMs M = 114.31 h, SD = 240.69; Controls M = 91.94 h, SD = 145.93; Declined M = 75.70 h, SD = 129.22); nor in the average number of ED presentations per patient (PRMs M = 1.31; Control M = 1.13; Declined M = 1.04).

#### CAU attendance

PRMs patients were significantly less likely to have ≥ 1 CAU visit compared to historical Controls (PRMs 20.8%, 10/48; Controls 31.8%, 20/63; Declined 14.8%, 4/27, p = 0.021), after adjusting for gender, age and disease stage. Similarly, the PRMs group had a significantly lower average number of CAU presentations per patient than Controls (multivariable negative binomial regression; M = 0.229 vs. M = 0.429; *p* = 0.035).

There was no significant relationship between the total number of breached items and the likelihood of visiting the CAU (mean of 16 breached items for CAU attendees versus 13 for non-attendees).

#### Allied health referrals

Across all assessments completed and breached (n = 90), 146 referrals were made to allied health services, most frequently for social work (25.3%), dietetics (18.5%), physiotherapy (18.5%) and occupational therapy (17.1%) (see Table 3).

**Table 3:** Referrals to allied health services in the PRM group

Allied health service ∞	Total number of referrals	Accepted n (%**)	Offered, but declined n (%**)
Social work	37	24 (64.9)	13 (35.1)
Dietetics	27	13 (48.1)	14 (51.9)
Physiotherapy	27	7 (26)	20 (74)
Occupational therapy	25	4 (16)	21 (84)
Clinical psychology	21	5 (23.8)	16 (76.2)
Palliative care	5	4 (80)	1 (20)
Lymphoedema clinic	2	2 (100)	–
Transport	1	–	1 (100)
Home care	1	–	1 (100)
Total	146	59 (40.4)	87 (59.6)

**Based on the total of referrals within each category

∞Multiple referrals can be made/attempted after a breach in a single action/phone call

A significantly higher proportion of PRMs patients (70.8%, n = 34/48) were offered ≥ 1 referral during the evaluation period compared to Controls (28.7%, n = 18/63, *p* < 0.0001). The PRMs group also had a significantly higher average number of referrals per patient than Controls (M = 1.27 vs. M = 0.38, *p* < 0.0001). Patients with more breached items were more likely to accept a referral (*p* = 0.0005). The likelihood of accepting a referral was not influenced by gender or stage of disease (gender *p* = 1; stage *p* = 0.779), or by patient’s age (*p* = 0.615).

#### Adoption

##### Feasibility of the implementation amongst HCPs

ORIC assessments were completed by HCPs, administrative and management staff, among others (baseline, n = 37; follow-up, n = 28; see demographics in Table 4). At baseline, organizational readiness for change (mean total ORIC score = 47.24, SD = 8.03), change commitment (M = 20.57 (SD = 3.51)) and change efficacy (M = 26.68, SD = 5.33) were all high, and repeated measures ANOVA (paired responses only), revealed no significant changes at follow-up.

**Table 4:** Demographic characteristics of baseline ORIC and HCP survey respondents

Characteristic	ORIC (n = 37)	HCP (n = 21)
Gender (%)		
Female	32 (86.5)	19 (90.5)
Male	5 (13.5)	2 (9.5)
Hospital (%)		
Hospital #1	26 (70.3)	11 (52.4)
Hospital #2	8 (21.6)	7 (33.3)
Hospital #3	3 (8.1)	3 (14.3)
Area of work (%)		
Allied health	13 (35.1)	13 (61.9)
Administration	12 (32.4)	NA
Cancer care coordination	6 (16.2)	6 (28.6)
Nursing	2 (5.4)	2 (9.5)
Management	2 (5.4)	NA
Other	1 (2.7)	NA

Twenty-one staff completed the baseline HCP surveys (see demographics, Table 4), and 17 completed follow-up HCP surveys. Only paired responses (n = 17) were included in the nominal symmetry test. Significant changes were detected in 2/18 items at follow-up, with more staff reporting a higher level of confidence when asking patients to complete assessments (64.7% at baseline vs. 88.2% at follow-up, *p* = 0.0046), and when describing the assessment tool to patients (64.7% at baseline vs. 76.47% at follow-up, *p* = 0.0018). See Additional file 2 for all other results.

##### Change agents implementing ePRO

The change agents implementing PRMs included lung cancer care coordinators (n = 4/4), PRMs champions including administrative, management and nursing staff who facilitated patient onboarding (n = 6/6), medical oncologists (n = 6/11), and radiation oncologists (6/9), resulting in 73% (n = 22/30) total change agents.

#### Implementation

##### Number and proportion of patients screened

At Hospital #1, there were 32 eligible new lung cancer patients in the 5-month evaluation period, of whom 87.5% (n = 28) were screened, 43.8% (n = 14) were onboarded, 43.8% (n = 14) declined and 12.5% (n = 4) were systematically missed. At Hospital #2, all 34 (100%) eligible patients were screened and 70.6% (n = 24) were onboarded, 29.4% (n = 10) declined. At Hospital #3, all 13 (100%) eligible patients were screened, 76.9% (n = 10) were onboarded and 23.1% (n = 3) declined.

##### Clinical alerts actioned

Every completed assessment (n = 90) had breached items; 95.6% (n = 86) were actioned by HCPs.

##### Number of cancer staff receiving orientation

Across the three hospitals, 78 staff received orientation and/or training and implementation resources (training materials, user guides). Trained staff included allied health professionals (n = 27), administrative and management staff (n = 22), care coordinators (n = 7), radiation oncologists (n = 6), medical oncologists (n = 6), nurses (n = 5), IT Specialists (n = 2), pathologist (n = 1), radiation therapist (n = 1). The PRMs webpage housing PRMs resources, accessible by any hospital staff, had 52 views.

#### Maintenance

##### Staff maintaining engagement

The model of care adopted for implementation was driven by the care coordinators [33], all of whom (n = 4, 100%) maintained engagement over the implementation period, through onboarding eligible patients, assisting patients to complete assessments as required, reviewing the PRMs reports and actioning clinical alerts for breached assessments.

More broadly, in the HCP follow-up survey, more than a third (35%, 6/17) reported discussing the PRMs assessment results with a few, most, or all of their lung cancer patients. Furthermore, more than a third (35%, 6/17) said yes or sometimes to reviewing the care recommendations provided for above-threshold scores on the assessments. However, it is worth noting that the HCP survey participants included allied health professionals (n = 10), care coordinators (n = 5), and nurses (n = 2), and the questions were most relevant to care coordinators and nurses.

##### Staff feedback on PRMs implementation

The main themes generated from staff interviews and surveys, and field notes, centred on: (a) benefits of PRM implementation, including perceived improvements in patient care; (b) how to engage non-English speaking patients; (c) the role of care coordinators and the extent of clinicians’ engagement; and (d) value of extending this model to all cancers. Illustrative HCP quotes are included in Table 5.

**Table 5:** Staff quotes regarding the PRMs system

A	*“I have just seen another patient today with a phone interpreter who has scored red in several categories which I did not pick up on during the consultation. There is so much to go through in a medical consultation that clearly, we miss out on evaluating the psychosocial and practical concerns of the patient, unless they bring it up themselves. Clearly, I have not been doing this well for previous patients. Hooray for PROMs”* (L046, radiation oncologist) *“I think it [the PROMs system] addresses a very important gap that we have discussed previously, and I think it helps both the doctors and the patients at trying to narrow that gap. ….. at least they [patients] feel like they have been heard.”* (B011, medical oncologist) *“There’s been more appropriate referrals. So, yeah… I think sometimes like I said social work could be a very grey area… whereas this [the care coordinator referral] has very specific reasons for why social work would need to be involved.”* (L054, social worker) *“…we do have more contact with patients.”* (L007, care coordinator)
B	“*Personally, it is what we have been doing anyway, so it’s formalizing it. From my point of view, it’s more registering the importance of that and making the patient realize that they can concentrate by doing the PROMs on questions that incidentally might happen when the patient is with the consultant, so they feel more relaxed and that everybody is working together with them and that they are the centre of care which is how everything should be.*” (L003, care coordinator)
C	*“I think it’s really difficult [using ePROs with non-English speaking population and referring them to allied health service] because each cultural group has different intricacies about what they find acceptable or not with medical care and how open they are to receive care…”* (L056, radiation oncologist) *“CALD [culturally and linguistically diverse] care coordinators would be a great idea… It’s difficult dealing with interpreters.”* (L046, radiation oncologist) *“The care coordinator CALD model will be very important to understand language and culture.”* (L058, information technology staff)
D	*“… the best thing is not to onboard in clinic; it’s better to do it over the phone.”* (L007, care coordinator) *“It’s better if the patient can complete the survey from home…. the iPad is not practical.”* (B008, care coordinator) *“Patients on maintenance or survivorship—we need to add another booking [queued appointment to complete the survey] and then you could stretch it out probably every 3 months.”* (L003, care coordinator) *“What I find though is that patients’ feedback (not all of them) but some find the constant needing to do a survey… a bit too … intense for them. It depends on how well they [patients] are… those ones with a lot of problems, they [patients] sometimes just reported it and obviously you don’t address every single one [issue] at the same time but more in terms of priorities. And then not long after they are asked the same thing again, and you haven’t even sorted out all the others…”* (C009, medical oncologist)
E	*“I think I probably didn’t use it as much as I should have, but I have used the tab. It wasn’t any extra burden. I thought it was good having [care coordinator name] there, she would just email me and flag if there was something that hadn’t been addressed for the patient… she would email me and say ‘hey, this came up from the PROMs. Could you please talk about it and address it?’… I think most issues were resolved with that [referring to the communication with care coordinator].”* (L056, radiation oncologist) *“I read the notes [from the care coordinator] before the appointment… It [the CINSW PRMs system] does fill the gap.”* (B011, medical oncologist)
F	*“[Care coordinator name] has been great. She names the reason of the referral in the referral form. The care coordinator has been critical. It is an entrance point [the work of the care coordinator]… then I am able to address that. It is a point of engagement… I always have ongoing conversations with the care coordinator to discuss patients.”* (L054, social worker)
G	“*Doctors are still unaware of the whole concept. So, lack of support from them. Not acknowledging PRMs at the time of consult and discuss the concerns from that while seeing the patient leading to mistrust of the patients and carers continuing the future surveys.*” (B008, care coordinator) *“We need the clinicians to understand they need to check the PROMS… clinicians need to acknowledge it, open the report when sitting with the patient.”* (L003, care coordinator)
H	*“It is applicable if we have sufficient care coordinators for all tumour sites. …It comes back down to the resources because [it] is very labour and resource intensive. It’s all good that you get them [the PROMs] to identify what’s their problems, but then what are you going to do about them? And I guess it’s also trying to address those issues requires more people. Sometimes, you need a psychologist, but then the psychologist can only just handle so much, and we only got part-timers. So, if you’ve got unlimited amount of resources and money, then it would be ideal…”* (C009, medical oncologist) *“We make the referrals as soon as they have done the PROMs… so we make the referral to whatever they need to go. But I guess… how long does it take for that referral to get picked up?”* (L007, care coordinator)
I	*“It’s been quite a holistic form of providing care for people. It’s more of a team way of approach for the patient and their care.”* (L054, social work)

##### Benefits of PRM implementation

Some participants perceived that the PRMs assessments significantly improved their clinical service and increased patient contact (Table 5, section A). Others thought that using the tool in routine care formalizes their current practice while improving the patient experience (Table 5, section B).

##### Engaging non-English speaking patients

Staff highlighted challenges engaging non-English speaking patients and made suggestions regarding implementing PRMs with these patients and those with low digital literacy (Table 5, section C). Some improvements in procedures were recommended, including patient onboarding and tailoring assessment frequency (Table 5, section D).

##### Care coordinator role and clinician engagement

Care coordinators were perceived as integral to successful delivery of PRMs-informed care by oncologists (Table 5, section E) and allied health staff (Table 5, section F). However, while doctors largely relied on care coordinator notes to prioritize patients’ concerns, care coordinators perceived a greater need for clinicians to engage and acknowledge PRMs assessment reports during the clinical consultation, to facilitate patient engagement (Table 5, section G).

##### Extending this model of care

Most staff strongly endorsed the system being broadly implemented across all tumour sites, with doctors in particular suggesting that it would make it easier for clinicians, who would get used to expecting to see assessments from ALL patients, rather than a sub-group. However, the importance of the model being adequately resourced was unanimously reiterated, particularly with tumour-specific care coordinators and allied health services to deal with increased referrals (Table 5, section H). Implementation across the whole cancer service was also perceived to support a more holistic, team-based model of care (Table 5, section I).

## Discussion

To leverage the established benefits of utilizing ePROMs in routine clinical care, we drew on an implementation science approach using the RE-AIM framework to plan and evaluate the PRMs system implementation in the routine care of lung cancer patients. These patients are often older, with co-morbidities, commonly diagnosed at an advanced stage, and with a complex array of supportive care needs, hence the imperative for readily identifying and addressing their needs [39].

Our results suggest that the PRMs system was overall successfully implemented into routine care, with a high level of *reach* in the target population, and with measurable *effect* in increased allied health referrals and fewer PRMs patients attending the CAU than control patients. The results suggest timely response to the early identification of patients’ concerns. Staff engagement in PRMs training activities was high, and likely associated with improved confidence when asking patients to complete assessments and when describing the assessment tool to patients (*adoption*, *implementation*). Staff supported extending the PRMs system to all tumour groups, provided adequate care coordinator and allied health resources were available.

This study contributes important new knowledge to understanding how to translate effective interventions into routine care and address some of the specific local challenges arising from implementation processes. In preparation for implementation, we had conducted a clinic audit to understand our specific population’s capabilities for completing PRMs assessments. Despite two-thirds of patients in the audit reporting being capable of completing assessments in English [27], PRM implementation revealed that almost half (46%) of the target population were deemed ineligible due to lower levels of literacy, predominantly due to language. Implementation sites had large non-English speaking populations, and the availability of English-only assessments at the time of implementation meant the majority of this target group were not onboarded unless they were assisted by family or interpreters. A multi-language PRMs system is in development to extend the *reach* of PRM implementation. However, the results highlight the importance of other teams undertaking implementation projects to fully understand their local contexts and populations, to “leave no one behind” and avoid widening existing disparities [40].

Measuring the effect of implementation requires careful thought about the relevant outcomes and timeframe for detecting change. Our published trial of the ePROM system [17] reported 33% significantly fewer ED visits in the intervention group. While this implementation study failed to detect a similar finding, potentially due to a limited 5-month evaluation period (tied to funding timeline) and limited sample size, we observed other effects indicative of an intervention impact. Compared to historical controls, PRMs patients received significantly more allied health service referrals and had fewer CAU attendances. These two results are likely inter-related, with the higher rate of allied health referrals potentially reducing the need for CAU visits, suggesting that early management of symptoms may mitigate the escalation of toxicities [41] and supporting the *effect* of implementation.

As part of evaluating *adoption*, understanding organizational readiness for change before PRMs implementation in routine care is critical, as staff in organizations with higher readiness for change are more likely to initiate change, be collaborative and cooperative, and willing to implement new evidence-based practices [42, 43]. With staff at two of the three hospitals having been familiarised with ePROMs during our previous trial [17], it is unsurprising that the baseline level of organizational readiness for change was high, with little shift throughout implementation. Furthermore, rigorous stakeholder engagement activities resulted in a high rate of relevant staff (73%) being implementation change agents, aligning with the literature highlighting stakeholder engagement [44] and use of change agents or clinical champions [45, 46] as important implementation strategies. Our results support the importance of carefully selecting initial implementation sites with greater readiness for change and clinical champions, as these become influential when engaging new sites. In the local context, the remaining hospitals in SWSLHD requested the PRMs system as soon as feasible after seeing the level of support from initial sites (personal communication).

Our trial [17] supported PRMs implementation driven by care coordinators, which is consistent with the majority of published ePROM trials reporting nursing staff reviewing and actioning ePROM results [47–50], compared to only a few studies referring to doctors [51]. Implementation success was facilitated by including the lung cancer care coordinators in the implementation planning, ensuring a functional and efficient workflow was developed for onboarding patients, reviewing assessment results, making referrals, triaging and providing direct care, as required. Coordinators were critical “filtering agents” for oncologists, reviewing PRM reports and highlighting specific issues to address during patient consultations. This process was highly valued by doctors, for whom it was a significant time-saver. Implementation success could have been further enhanced if the doctors made more explicit reference to PRMs reports during consultations. Coordinators perceived this lack of explicit acknowledgement as resulting in some patients losing interest in completing assessments. Our findings are consistent with a UK trial showing that most doctors (63%) did not explicitly refer to PROMs data and patients perceive their doctors to have greater knowledge than nurses [52]. Doctors’ acknowledgement of ePROMs results could potentially improve patient engagement levels and should be included in training/orientation processes in future implementation projects.

Staff engagement strategies, including PRMs orientation, training and re-training in accessing PRM reports, appeared to have a significant impact on staff confidence at follow-up, when describing and asking patients to complete the ePROM tool. This is consistent with evidence about the importance of supportive strategies such as staff education [37]. Furthermore, staff perceived the PRMs system as beneficial to patient care, strongly endorsing its implementation across all tumour groups. It aligns with systematic review evidence and meta-synthesis that oncology clinicians’ perception of the value of the intervention may promote adoption [53]. The evaluation data suggests that the PRMs system was successfully *adopted* by staff at participating hospitals.

Our PRMs system was successfully implemented at all three hospitals, with high levels of patients onboarding, review and actioning of clinical alerts. Two of the three hospitals successfully onboarded all eligible patients (100%); and nurses demonstrated an even higher rate of reviewing and actioning clinical feedback reports (95.6%) in routine care than previously achieved in research settings [17, 50]. The response to clinical alerts included significantly more referrals to allied health services in PRMs versus historical control groups. Importantly, the referral recipients, allied health staff, highlighted that they received more appropriate referrals and that the PRMs reports helped inform care delivery.

The COVID-19 pandemic period, especially the community lockdowns, highlighted the importance of collecting routine PRMs from patients. The lack of face to face consultations during this period are likely to have reduced clinical staff’s ability to detect distress or other psychological issues in patients via their usual consultations methods. During this period patients expressed a strong desire to retain more remote consultations rather than the inconvenience, infection risk and cost of face to face consultations. It was thought that in the future PRMs could actually support a move to more remote consultations with patients even in periods without community lockdown.

Whilst five months is insufficient to assess whether the PRMs system was successfully *maintained* over time, the RE-AIM framework guided planning to increase likely maintenance. The staff interviews provided important contextual insights to support ongoing maintenance, particularly the need for adequate human resource infrastructure of care coordinators and allied health staff. This is consistent with systematic review evidence that resource availability enables successful PRMs implementation and sustainability [24]. Staff recommended other strategies to support sustained implementation, including refinements to onboarding procedures for first and subsequent ePROMs, and tailoring ePROM survey frequency to patient need. As oncology care settings learn from their experience, the use of the PRMs system will continually optimize the timing of administering ePROMs [54].

## Study limitations

Participating hospitals were based in a community with a high proportion of the population born overseas (43%), and almost half (45%) speaking a language other than English at home [28]. Since the PRMs system was only available in English, the sample excludes a sizable proportion of the local community. This study may have benefited from patient feedback regarding the system-level changes they experienced, to compare their views with those from healthcare professionals. The COVID-19 pandemic impacted some outcomes, including ED presentations. Other impacts on implementation effectiveness are unclear, such as visits to the CAU.

## Conclusion and practical implications

Our study demonstrates the potential of the PRMs system to enhance the routine care of lung cancer patients, and the value of using the RE-AIM framework for implementation planning and defining outcomes to measure implementation success in a real-world setting. Critical success factors were developing multi-faceted evidence-based implementation strategies that were tailored to local contexts, as was large-scale engagement and training of stakeholders. These factors are recommended for adoption for future PRMs implementation initiatives. Successful PRMs system implementation in routine care could pave the way for redefining models of care that leverage the capabilities of automated web-based strategies and engaging staff across multiple disciplines in implementation processes [55].

## Supplementary Information

**Additional file 1:** Healthcare provider survey.

**Additional file 2:** Health care provider survey - Responses to individual items at two time points.

## Data Availability

De-identified data that supports the findings of this study are available upon reasonable request to the corresponding author and approval from the South Western Sydney Local Health District ethics committee.
