# Supplementary material for: Stepping into the real world: a mixed-methods evaluation of the implementation of electronic patient reported outcomes in routine lung cancer care
Source: J Patient Rep Outcomes. 2022 Jun 20;6:70. doi: 10.1186/s41687-022-00475-6 (PMC9207870; doi:10.1186/s41687-022-00475-6)
Supplement: Supplementary file 1 — Additional file 1: Healthcare provider survey. [file 41687_2022_475_MOESM1_ESM.docx]

**Additional file 1**

**Healthcare Provider Survey**

**About PROMPT-Care tool use**

The following questions are in relation to the delivery of the PROMPT–Care electronic screening tool. Please read the statements carefully in each part and select the answer that applies to you most.

**Part A**

We would like to know if you feel there will be any potential barrier(s) in delivering the PROMPT-Care screening tool.

To what extent do you think the following factors may make it difficult to administer the PROMPT-Care screening tool?

- The physical environment in which the tool is delivered.
- The amount of time required to deliver the tool.
- Understanding the benefit of the tool.
- Understanding the reason why the tool needs to be implemented.
- Belief in the need for this change.
- The time point of the patient’s journey in which the tool is administered.
- Lack of education about psychosocial concerns.
- Lack of skills in dealing with psychosocial concerns which patients may experience.

**Response options: Not at all, A little, Neutral, To some extent, Very much.*

Please list any other barriers that you feel you may encounter when using the PROMPT-Care screening tool.

**Part B**

We would like to understand your knowledge and attitudes surrounding the use of the PROMPT–Care screening tool.

To what extent do you agree with each of the following statements?

- I know the tool is useful in identifying psychosocial problems.
- I know why psychosocial screening is recommended for all patients presenting to cancer care.
- I am familiar with the content of the different screening tools (Distress Thermometer/Patient Checklist & Edmonton Symptom Assessment System (ESAS-revised) used within the PROMPT-Care tool.
- I understand the purpose of each of the sections in the PROMPT–Care tool.

*Response options: Strongly disagree, Somewhat disagree, Neither agree nor disagree, Somewhat agree, Strongly agree.*

**Part C**

How confident do you feel about your skills for each of the following aspects of patient care?

Please indicate your level of confidence about:

- Describing the PROMPT-Care tool to the patient.
- Asking the patient to complete the PROMPT-Care tool.
- Delivering the PROMPT-Care screening tool to patients in clinic at their first appointment.
- Educating patients about the importance of patient reported outcomes as part of their cancer care.
- Discussing psychosocial issues with patients.
- Recognizing signs of anxiety/depression.

**Response options: Not at all confident, Not confident, Neutral, Somewhat confident, Extremely confident, Not relevant to my job*

**Part D (only in follow-up survey)**

We would like to understand your overall use of the PROMPT–Care screening tool. How important is this in your role?

- Using the PROMPT-Care screening tool
- Patients using the PROMPT-Care screening tool to communicate their issues.
- Referring to other allied health services such as dietetics, physiotherapy, social work or psychology.
- Discussing overall concerns with patients.
- My organization (e.g. hospital, clinic) recognizing my role in providing the PROMPT-Care screening tool to support our patients.
- My work colleagues recognizing my role in providing the PROMPT-Care screening tool to our patients.
- Other members of the health care team recognizing my role in providing the PROMPT-Care screening tool to our patients.

*Response options: Not important, Slightly important, Neutral, Important, Very important.*

**Other questions (only in follow-up survey)**

In the past 3 to 4 months, about what proportion of your **lung cancer patients** did you discuss their PROMPT-Care survey results with?

- None
- A few of them
- About half
- Most of them
- All of them

Do you currently review the care recommendations provided for above-threshold scores on the PROMPT-Care screening tool?

- Yes
- No
- Sometimes

Please specify the reason for your answer

In your opinion, do you feel it is your role to address the recommendations provided by the PROMPT- Care tool?

- Yes
- Yes, but I don’t feel I have the skills to do that
- No
- Maybe
