# Supplementary material for: Stepping into the real world: a mixed-methods evaluation of the implementation of electronic patient reported outcomes in routine lung cancer care
Source: J Patient Rep Outcomes. 2022 Jun 20;6:70. doi: 10.1186/s41687-022-00475-6 (PMC9207870; doi:10.1186/s41687-022-00475-6)
Supplement: Supplementary file 2 — Additional file 2: Health care provider survey - Responses to individual items at two time points. [file 41687_2022_475_MOESM2_ESM.docx]

**Additional file 2**

Responses to individual items

| **Questionnaire statements** | **T1** | **T2** | **Significance****  **(*p* value=)** |
| --- | --- | --- | --- |
|  | **%(n*)** | **%(n*)** |  |
| PART A: To what extent have the following factors made it difficult to administer the PROMPT-Care screening tool? |  |  |  |
| The physical environment in which the tool is delivered |  |  | 0.07 |
| *Has not made it difficult* | 29.4 (5) | 17.65 (3) |  |
| *Neutral* | 29.41 (5) | 64.71 (11) |  |
| *Has made it difficult* | 41.18 (7) | 17.65 (3) |  |
| The amount of time required to deliver the tool |  |  | 0.24 |
| *Has not made it difficult* | 29.4 (5) | 11.7 (2) |  |
| *Neutral* | 29.41 (5) | 58.8 (10) |  |
| *Has made it difficult* | 41.18 (7) | 29.41 (5) |  |
| Understanding the benefit of the tool |  |  | 0.75 |
| *Has not made it difficult* | 47.06 (8) | 52.9 (9) |  |
| *Neutral* | 29.4 (5) | 35.3 (6) |  |
| *Has made it difficult* | 23.5 (4) | 11.7 (2) |  |
| Understanding the reason why the tool needs to be implemented |  |  | 0.97 |
| *Has not made it difficult* | 41.2 (7) | 47 (8) |  |
| *Neutral* | 41.2 (7) | 35.3 (6) |  |
| *Has made it difficult* | 17.6 (3) | 17.6 (3) |  |
| Belief in the need for this change |  |  | 0.91 |
| *Has not made it difficult* | 29.4 (5) | 41.2 (7) |  |
| *Neutral* | 47 (8) | 41.2 (7) |  |
| *Has made it difficult* | 25.5 (4) | 17.6 (3) |  |
| The time point of the patient’s journey in which the tool is administered |  |  | **0.04** |
| *Has not made it difficult* | 29.4 (5) | 41.2 (7) |  |
| *Neutral* | 23.5 (4) | 52.9 (9) |  |
| *Has made it difficult* | 47 (8) | 5.8 (1) |  |
| Lack of education about psychosocial concerns |  |  | **0.05** |
| *Has not made it difficult* | 29.4 (5) | 35.3 (6) |  |
| *Neutral* | 23.5 (4) | 52.9 (9) |  |
| *Has made it difficult* | 47 (8) | 11.7 (2) |  |
| Lack of skills in dealing with psychosocial concerns which patients may experience |  |  | 0.05 |
| *Has not made it difficult* | 35.3 (6) | 35.3 (6) |  |
| *Neutral* | 23.5 (4) | 52.9 (9) |  |
| *Has made it difficult* | 41.2 (7) | 11.7 (2) |  |
| Part B: To what extent do you agree with each of the following statements? |  |  |  |
| I know the tool is useful in identifying psychosocial problems |  | | 1 |
| *Disagree* | 5.8 (1) |  |  |
| *Neutral* | 11.7 (2) | 11.7 (2) |  |
| *Agree* | 82.3 (14) | 88.2 (15) |  |
| I know why psychosocial screening is recommended for all patients presenting to cancer care |  |  | **0.05** |
| *Disagree* | 5.8 (1) | - |  |
| *Neutral* | 5.8 (1) | - |  |
| *Agree* | 88.2 (15) | 100 (17) |  |
| I am familiar with the content of the different screening tools (Distress Thermometer/Patient Checklist & Edmonton Symptom Assessment System (ESAS-revised) used within the PROMPT-Care tool |  |  | 0.31 |
| *Neutral* |  | 5.8 (1) |  |
| *Agree* | 100 (17) | 94.1 (16) |  |
| I understand the purpose of each of the sections in the PROMPT–Care tool |  |  | NA |
| *Agree* | 100 (17) | 100 (17) |  |
| PART C: How confident do you feel about your skills for each of the following aspects of patient care? |  |  |  |
| Describing the PROMPT-Care tool to the patient |  |  | **0.0018** |
| *Not confident* | - | 5.8 (1) |  |
| *Neutral* | 17.6 (3) | 5.8 (1) |  |
| *Confident* | 76.47 (13) | 88.4 (15) |  |
| *Not relevant to my job* | 5.8 (1) |  |  |
| Asking the patient to complete the PROMPT-Care tool |  |  | **0.0046** |
| *Not confident* | - | 5.8 (1) |  |
| *Neutral* | 23.5 (4) | 5.8 (1) |  |
| *Confident* | 64.71 (11) | 88.2 (15) |  |
| *Not relevant to my job* | 11.7 (2) | - |  |
| Delivering the PROMPT-Care screening tool to patients in clinic at their first appointment |  |  | 0.23 |
| *Not confident* | 23.5 (4) | 11.7 (2) |  |
| *Neutral* | 29.4 (5) | 11.7 (2) |  |
| *Confident* | 35.3 (6) | 76.5 (13) |  |
| *Not relevant to my job* | 11.7 (2) | - |  |
| Educating patients about the importance of patient reported outcomes as part of their cancer care |  |  | 0.88 |
| *Neutral* | 17.7 (3) | 11.7 (2) |  |
| *Confident* | 70.6 (12) | 88.2 (15) |  |
| *Not relevant to my job* | 11.7 (2) | - |  |
| Discussing psychosocial issues with patients |  |  | 0.39 |
| *Not confident* | 5.8 (1) | - |  |
| *Neutral* | 17.7 (3) | 5.8 (1) |  |
| *Confident* | 76.5 (13) | 94.1 (16) |  |
| Recognizing signs of anxiety/depression |  |  | 0.8 |
| *Not confident* | 5.8 (1) | - |  |
| *Neutral* | 5.8 (1) | 5.8 (1) |  |
| *Confident* | 88.2 (15) | 94.1 (16) |  |
| PART D: How important is this in your role?*** |  |  |  |
| Using the PROMPT-Care screening tool |  |  |  |
| *Not important or slightly important* | NA | 11.7 (2) | NA |
| *Neutral* | NA | 23.5 (4) |  |
| *Important or very important* | NA | 64.71 (11) |  |
| Patients using the PROMPT-Care screening tool to communicate their issues |  |  | NA |
| *Not important or slightly important* | NA | 5.8 (1) |  |
| *Neutral* | NA | 17.7 (3) |  |
| *Important or very important* | NA | 76.5 (13) |  |
| Referring to other allied health services such as dietetics, physiotherapy, social work or psychology |  |  | NA |
| *Neutral* | NA | 11.7 (2) |  |
| *Important or very important* | NA | 88.2 (15) |  |
| Discussing overall concerns with patients |  |  | NA |
| *Neutral* | NA | 11.7 (2) |  |
| *Important or very important* | NA | 88.2 (15) |  |
| My organization (e.g. hospital, clinic) recognizing my role in providing the PROMPT-Care screening tool to support our patients |  |  | NA |
| *Not important or slightly important* | NA | 29.4 (5) |  |
| *Neutral* | NA | 29.4 (5) |  |
| *Important or very important* | NA | 41.2 (7) |  |
| Note: some response options (i.e. disagree, neutral, etc) were not presented as they were not selected at any timepoint.  *Out of 17 respondents  **Chi-Square *p* value  ***Items on follow-up survey only | | | |
